# Supplementary material for: The Only Chemoreceptor Encoded by che Operon Affects the Chemotactic Response of Agrobacterium to Various Chemoeffectors
Source: Microorganisms. 2021 Sep 10;9(9):1923. doi: 10.3390/microorganisms9091923 (PMC8466855; doi:10.3390/microorganisms9091923)
Supplement: Supplementary file 1 [file microorganisms-09-01923-s001.zip › microorganisms-1358834-supplementary.pdf]

A. Schematic diagram of gene replacement strategy (First cross occurs in upstream)

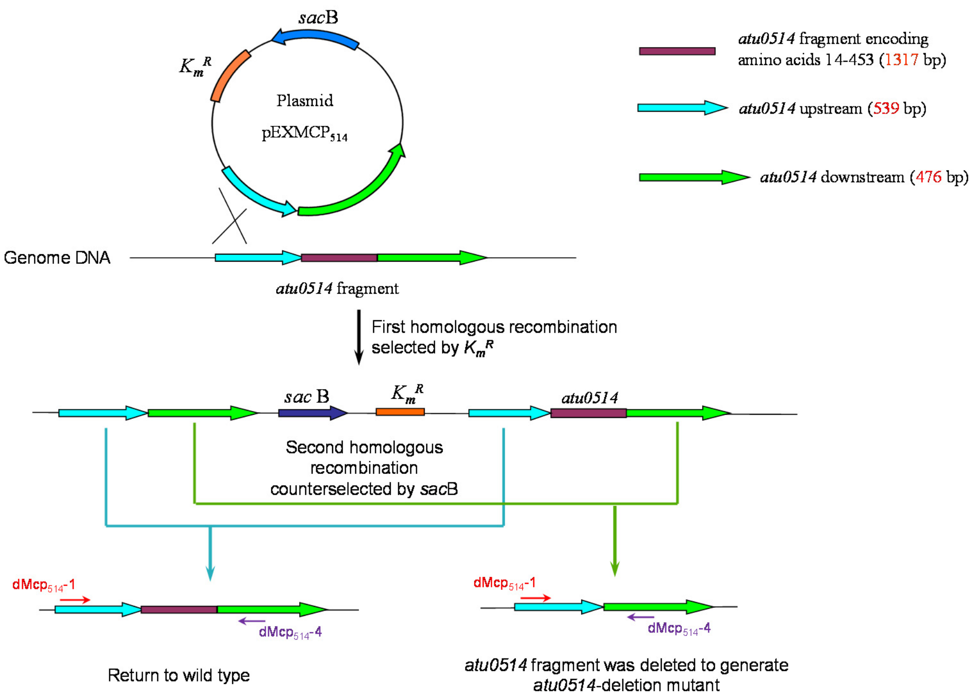

B. Screening of *atu0514*-deletion mutant.

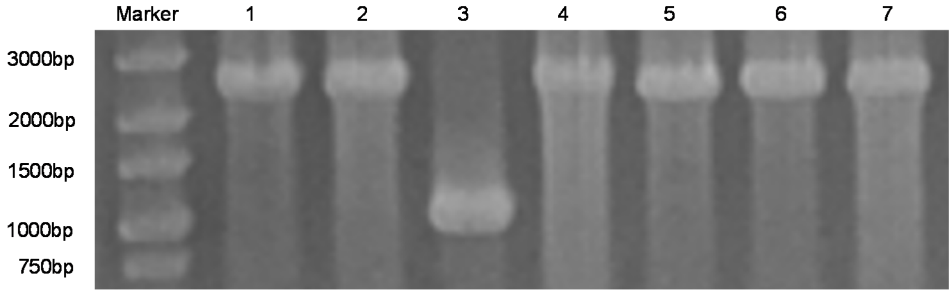

C. Sequencing result of PCR fragment amplified from the mutant.

```
Δ514    1 CCGTTGATTTGTTTTAGGGGAACCCGCACGCGCTTGTCCAGCGCGCAGGCCGGAAAAACCGGCCGGAGCGTCCGATTCCTTTTCGTTTTTGCCCGTTTGC 100
C58     1 CCGTTGATTTGTTTTAGGGGAACCCGCACGCGCTTGTCCAGCGCGCAGGCCGGAAAAACCGGCCGGAGCGTCCGATTCCTTTTCGTTTTTGCCCGTTTGC 100

Δ514  101 GGCAAAGTAAGGGCGACAATGGCTAAAAGCTTTTACAAATTTTACCGTTTGAGGCCGGTGCTTATGTTTCTTAACTTTCAAGCAAGGAATTAACCATAAA 200
C58   101 GGCAAAGTAAGGGCGACAATGGCTAAAAGCTTTTACAAATTTTACCGTTTGAGGCCGGTGCTTATGTTTCTTAACTTTCAAGCAAGGAATTAACCATAAA 200

Δ514  201 GATTGGTGGTATTTCCACGGCGGAATGGGGTTCCTCCCCTTCCAGGCATGACGCCGCACTGCCGTACCGGGCTCGATCCGGTATTTTCGTTTCTTGGTTC 300
C58   201 GATTGGTGGTATTTCCACGGCGGAATGGGGTTCCTCCCCTTCCAGGCATGACGCCGCACTGCCGTACCGGGCTCGATCCGGTATTTTCGTTTCTTGGTTC 300

Δ514  301 AAGAAGGTCTGGCAAGCGTCGATGCCTCATCGTCCACGATGGGTATGTTCGTTCAAAAATCCCTGGAGGGTGCTGGCGTTTTGAACCTTGGCCAATTGGC 400
C58   301 AAGAAGGTCTGGCAAGCGTCGATGCCTCATCGTCCACGATGGGTATGTTCGTTCAAAAATCCCTGGAGGGTGCTGGCGTTTTGAACCTTGGCCAATTGGC 400

Δ514  401 CGGGTTTTACCGGCCATCATGTTGTTGGGGACGTCGGTTGGGGCACGATTTGCCATCAGATCAAGCGCGTAGATCGGCAGGCGGAAGCCTGGCAGGACGG 500
C58   401 CGGGTTTTACCGGCCATCATGTTGTTGGGGACGTCGGTTGGGGCACGATTTGCCATCAGATCAAGCGCGTAGATCGGCAGGCGGAAGCCTGGCAGGACGG 500

Δ514  501 TTGCGCTTTGCCGGCCTTGATGAGATGCAATCCGATTT--------------------------------------------------------------- 538
C58   501 TTGCGCTTTGCCGGCCTTGATGAGATGCAATCCGATTTCCTGCGCAATTATCGCGGCATGCTGGAGCCTTACGTCAAGGCCGGCCTGCGCGACGTGATGA 600

Δ514  540 -----------------------------------------------------------GATGCGCTGCACACCGTCATTCTGGAACTCGGCCGCACCGT 580
C58  1801 AACAGCAACGGCAGGCCGGGCGGAATGTCGCCGACATGGGCGCCAGCGCCTCCGAGGCCGATGCGCTGCACACCGTCATTCTGGAACTCGGCCGCACCGT 1900

Δ514  581 GCGCGCGTTCCGTATTGCCCGCCAGGAACATTTCGCTGCCGCCGCCGGTGCGGACTGGCAGCACTCATCATTTATGGCCCGTGGGGATAACCGCACGGAT 680
C58  1901 GCGCGCGTTCCGTATTGCCCGCCAGGAACATTTCGCTGCCGCCGCCGGTGCGGACTGGCAGCACTCATCATTTATGGCCCGTGGGGATAACCGCACGGAT 2000

Δ514  681 AACAGCCAGAATTACCAACAATTCAGACGTCAGGGAGTCATGTAATGGCAGCCAGAAAAGCAGCTGAGGCGACGCTGAAACTGTCACCGGTGCTGGATCT 780
C58  2001 AACAGCCAGAATTACCAACAATTCAGACGTCAGGGAGTCATGTAATGGCAGCCAGAAAAGCAGCTGAGGCGACGCTGAAACTGTCACCGGTGCTGGATCT 2100

Δ514  781 CAACGAAGCATCGGCTCTGCACGGCAAGCTGATGACGCTGAGAGGAGCACCTTTGTCGGTCGATGCGTCCGAGGTGGAGCGCATTGGCGCGCTTTGCGCC 880
C58  2101 CAACGAAGCATCGGCTCTGCACGGCAAGCTGATGACGCTGAGAGGAGCACCTTTGTCGGTCGATGCGTCCGAGGTGGAGCGCATTGGCGCGCTTTGCGCC 2200

Δ514  881 CAGGTCCTCATGGCCGGCGCGAAATCCTGGGAAGAGGATGGCAAGCCCTTCGGTTTCGCAAGGGTTTCCGATGCATTCGACAAGACCATGAAACTGATAG 980
C58  2201 CAGGTCCTCATGGCCGGCGCGAAATCCTGGGAAGAGGATGGCAAGCCCTTCGGTTTCGCAAGGGTTTCCGATGCATTCGACAAGACCATGAAACTGATAG 2300

Δ514  981 GCGTCGATATCGACCATTTGCTCCCTAAGGAGATG 1015
C58  2301 GCGTCGATATCGACCATTTGCTCCCTAAGGAGATG 2335
```

Figure S1. Construction procedure, screening and verification of *atu0514*-deficient mutants. (**A**) Schematic diagram of gene replacement strategy. The suicide plasmid $pEXMCP_{514}$ carrying the upstream (light blue arrow) and downstream (green arrow) sequences of *atu0514* was introduced to the recipient cell. Because this plasmid can not be replicated in *A. fabrum*, only the transformants that the plasmid was integrated into the genome DNA at the locus of the target gene by homologous recombination can grow under the selection pressure of kanamycin. The K_m^R (orange rectangles) represents kanamycin-resistant gene. The *sac*B (blue arrows) is a counterselectable marker gene (sucrose suicide gene) that confers sucrose sensitivity. A fragment of *atu0514* was shown in dark red rectangles. Colonies having undergone single cross-over homologous recombination (×) were screened as kanamycin resistance and sucrose sensitivity. Second cross-over homologous recombination event was selected by plating the single cross-over colonies on media containing 5% sucrose. The *atu0514*-deletion (or replacement) mutants were isolated from the kanamycin-sensitive and sucrose-resistant colonies. (**B**) Screening of *atu0514*-deletion mutants. The *atu0514*-deletion mutants were screened by PCR. Two oligonucleotides, $dMcp_{514}$-1 (annealed to upstream of *atu0514*) and $dMcp_{514}$-4 (annealed to downstream of *atu0514*), were used as the primers. PCR fragment amplified from wild type template is longer than that from mutant template. After agarose gel electrophoresis, only the PCR product in lane 3 was amplified from the *atu0514*-deletion mutant template. (**C**) Sequencing result of the PCR fragment amplified from the mutant. The red border rectangles indicate the rest fragment of *atu0514* and the black dot indicate the nucleotide shared by *atu0514* and *atu0515*.

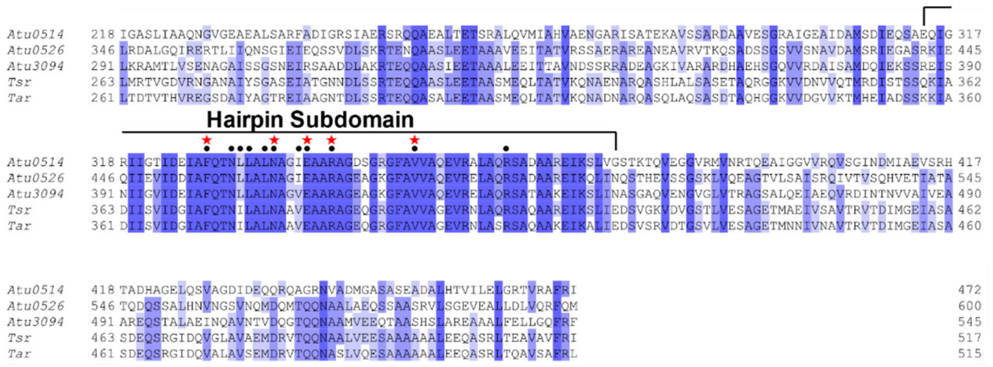

Figure S2. Sequences of three *A. fabrum* MCPs and two *E. coli* MCPs were aligned by Jalview. The numbers in two sides indicate the position of amino acid. The black dots represent the key residues of trimer contact in *E. coli* MCPs[52]. The red stars indicate the residues of the single residue substitution for MCP_{514}.

Table S1. Bacterial strains and plasmids used in this study

Bacterial strains	Relevant feature(s)	Reference/source
Escherichia coli		
E. coli DH5α	F–,ø80dlacZΔM15,Δ(lacZYAargF)U169,deoR,recA1,endA1,hsdR17(rK$^-$, mK$^+$), phoA, supE44, λ–, thi-1, gyrA96, relA1, for DNA cloning	Bethesda Research Laboratories
XL1 Blue	Report strain for Bacterial Two-hybrid System	Stratagene
Agrobacterium fabrum		
C58	Nopaline type strain; pTiC58, pAtC58	[63]
C58Δ514	C58 derivative deletional mutant in locus *atu0514*	This study
C58Δ514-C	C58Δmcp514 in which cheW1 expression was restored by pCBMCP$_{514}$	This study
C58Δ*w*	C58 derivative deletional mutant in locus both *chew*$_1$ and *chew*$_2$	[40]
C58Δ*a*	C58 derivative deletional mutant in locus *chea*	[40]
Δ514Δ*w*	C58 derivative triple-deletion mutant in locus *chew*$_1$, *chew*$_2$, and *atu0514*	This study
**Plasmid**		
pEX18Km	Derivative of pEX18Tc in which Tc$_r$ was replaced by *npt*III from pCB301; Km^R, Sur^S	[38]
pEXMCP$_{514}$	pEX18Km, carrying a 1015bp fragment, which consisted of 539bp upstream fragment (from -500 to +39) and 476bp downstream fragment (from +1359 to +1835) of *atu0514*	This study
pUCA-19	pUC19 carrying an agrobacterial replicon; Ap^R, Cr^R	[38]
pUCA-GMCP$_{514}$	pUCA-19, carrying an eGFP-MCP$_{514}$ expression cassette at HindIII and BamHI; Ap^R, Cr^R	This study
pUCA-GMCP$_{514}^{F328A}$	pUCA-19, carrying an eGFP-MCP$_{514}$ expression cassette with single residue-substution F382A at HindIII and BamHI; Ap^R, Cr^R	This study
pUCA-GMCP$_{514}^{F328P}$	pUCA-19, carrying an eGFP-MCP$_{514}$ expression cassette with single residue-substution F382P at HindIII and BamHI; Ap^R, Cr^R	This study
pUCA-GMCP$_{514}^{F328W}$	pUCA-19, carrying an eGFP-MCP$_{514}$ expression cassette with single residue-substution F382W at HindIII and BamHI; Ap^R, Cr^R	This study
pCB301	A minim binary vector plasmid, Km^R	[38]
pCBMCP$_{514}$	pCB301, carrying a 2207bp MCP$_{514}$ expression cassette at BamHI and EcoRI, which consisted of 662bp upstream and 1545bp orf of *atu0514*; Km^R	This study
pCBMCP$_{514}^{F328A}$	pCB301, carrying a 2207bp MCP$_{514}$ expression cassette with single residue-substution F382A; Km^R	This study
pCBMCP$_{514}^{N336A}$	pCB301, carrying a 2207bp MCP$_{514}$ expression cassette with single residue-substution N336A; Km^R	This study
pCBMCP$_{514}^{E340A}$	pCB301, carrying a 2207bp MCP$_{514}$ expression cassette with single residue-substution E340A; Km^R	This study
pCBMCP$_{514}^{R343A}$	pCB301, carrying a 2207bp MCP$_{514}$ expression cassette with single residue-substution R343A; Km^R	This study
pCBMCP$_{514}^{V353A}$	pCB301, carrying a 2207bp MCP$_{514}$ expression cassette with single residue-substution V353A; Km^R	This study
pBT	Bait plasmid, Cm^R	Stratagene
pBT-MCP$_{514}$MA	pBT, carrying *atu0514* orf at BamHI and XhoI, Cm^R	This study
pTRG	Target plasmid, Tc^R	Stratagene
pTRG-W$_1$	pTRG, carrying *chew*$_1$ orf at BamHI and XhoI, Tc^R	[40]
pTRG-W$_2$	pTRG, carrying *chew*$_2$ orf at BamHI and XhoI, Tc^R	[40]

References

63. Thomashow, M.; Nutter, R.; Montoya, A.; Gordon, M.; Nester, E. Integration and organization of Ti plasmid sequences in crown gall tumors. *Cell* **1980**, *19*, 729-739, doi:10.1016/S0092-8674(80)80049-3.

Table S2. Primers used in this study

Primers	Sequences	Purpose
dMCP$_{514}$-1	GACTCTAGAGGATCCCCGTTGATTTGTTTTAGGGGAAC	To amplify the upstream sequence of *atu0514* to construct pEXMCP$_{514}$
dMCP$_{514}$-2	GACGGTGTGCAGCGCATCGAAATCGGATTGCATCTCATCAAG	
dMCP$_{514}$-3	GAGATGCAATCCGATTTCGATGCGCTGCACACCGTCAT	To amplify the downstream sequence of *atu0514* to construct pEXMCP$_{514}$
dMCP$_{514}$-4	TGCTGCCAACTCGAGCATCTCCTTAGGGAGCAAATGGT	
egfp-MCP$_{514}$-1	GATTACGCCAAGCTTGATGGTGAGCAAGGGCGAGG	To amplify the sequence of *egfp*
egfp-MCP$_{514}$-2	GCTGCCACCTCCACCCTTGTACAGCTCGTCCATGCC	
egfp-MCP$_{514}$-3	GGTGGAGGTGGCAGCATGGGTATGTTCGTTCAAAAATC	To amplify the sequence of *atu0514* fusing to *egfp*
egfp-MCP$_{514}$-4	GGTACCCGGGGATCCTTACATGACTCCCTGACGTCTG	
pCBMCP$_{514}$-1	AGAACTAGTGGATCCCGAGGGTACCTGAAAGCCGAT	To amplify the complement sequence of pCBMCP$_{514}$
pCBMCP$_{514}$-2	CTTGATATCGAATTCTTACATGACTCCCTGACGTCTG	
F328A-1	GAGATCGCCGCCCAGACCAACCTTCTCGCCCT	To amplify the sequence of MCP$_{514}$ with the single residue-substution F328A
F328A-2	GGTTGGTCTGGGCGGCGATCTCGTCGATGGTGC	
F328P-1	GAGATCGCCCCCCAGACCAACCTTCTCGCCCT	To amplify the sequence of MCP$_{514}$ with the single residue-substution F328P
F328P-2	GGTTGGTCTGGGGGGCGATCTCGTCGATGGTGC	
F328W-1	GAGATCGCCTGGCAGACCAACCTTCTCGCCCT	To amplify the sequence of MCP$_{514}$ with the single residue-substution F328W
F328W-2	GGTTGGTCTGCCAGGCGATCTCGTCGATGGTGC	
N336A-1	TCGCCCTCGCCGCCGGCATCGAGGCG	To amplify the sequence of MCP$_{514}$ with the single residue-substution N336A
N336A-2	TGCCGGCGGCGAGGGCGAGAAGGTTGGTCT	
E340A-1	CCGGCATCGCCGCGGCACGGGCCG	To amplify the sequence of MCP$_{514}$ with the single residue-substution E340A
E340A-2	GTGCCGCGGCGATGCCGGCATTGAGGGCG	
R343A-1	GGCGGCAGCCGCCGGCGACAGCGGA	To amplify the sequence of MCP$_{514}$ with the single residue-substution R343A
R343A-2	CGCCGGCGGCTGCCGCCTCGATGCCG	
V353A-1	GGTTTCGCCGCCGTTGCGCAGGAAGTCCGGG	To amplify the sequence of MCP$_{514}$ with the single residue-substution V353A
V353A-2	TGCGCAACGGCGGCGAAACCGCGTCCG	
MCP$_{514}$MA-1	GGCCGCATCGAATTCTCGCTGATAGCCGCCCAG	To amplify the sequence of MCP$_{514}$ cytoplasmic singnal domain
MCP$_{514}$MA-2	TTAACTCGAGGATCCTTAGCGGGCAATACGGAACG	
